# Cognitive phenotype of psychotic symptoms in Alzheimer's disease: evidence for impaired visuoperceptual function in the misidentification subtype

**DOI:** 10.1002/gps.4265

**Published:** 2015-03-24

**Authors:** Suzanne J. Reeves, Chloe Clark‐Papasavas, Rebecca L. Gould, Dominic Ffytche, Robert J. Howard

**Affiliations:** ^1^ Department of Old Age Psychiatry, Institute of PsychiatryKing's College LondonSE5 8AFUK

**Keywords:** Alzheimer's disease, psychosis, subtypes, RVP, misidentification, ventral visual pathway

## Abstract

**Background:**

Establishing the cognitive phenotype of psychotic symptoms in Alzheimer's disease (AD) could localise discrete pathology and target symptomatic treatment. This study aimed to establish whether psychotic symptoms would be associated with poorer performance on neuropsychological tests known to correlate with striatal dopaminergic function and to investigate whether these differences would be attributed to the *paranoid* (persecutory delusions) or *misidentification* (misidentification phenomena +/− hallucinations) subtype.

**Methods:**

Seventy patients with probable AD (34 psychotic and 36 nonpsychotic) were recruited to the study. Analysis of covariance was used to compare motor speed and the rapid visual processing test of sustained visual attention, after adjusting for potential confounding factors. Multivariate analyses were used to compare performance across other cognitive domains. Significant findings were explored by separating patients on the basis of subtype.

**Results:**

Rapid visual processing performance accuracy was reduced in patients with psychotic symptoms (*F*_1,58_ = 5.94, *p* = 0.02) and differed significantly across subtypes (*F*_2,51_ = 3.94, *p* = 0.03), largely because of poorer performance in the misidentification compared with nonpsychotic group. Multivariate analyses (corrected for multiple comparisons) showed poorer performance on the incomplete letters task in psychotic patients (*F*_1,63_ = 8.77, *p* = 0.004) and across subtypes (*F*_2,55_ = 10.90, *p* < 0.001), similarly attributed to the misidentification subtype.

**Conclusions:**

These findings provide further support of the involvement of dopaminergic networks in the psychosis endophenotype in AD and, in addition, implicate the ventral (temporo‐occipital) pathway in the misidentification subtype. Future studies should investigate the early trajectory of neuropathological change *in vivo* across psychosis subtypes.© 2015 The Authors. *International Journal of Geriatric Psychiatry* published by John Wiley & Sons, Ltd.

## Introduction

Psychotic symptoms (delusions and hallucinations) are highly prevalent in people with Alzheimer's disease (AD) (Ropacki and Jeste, 2005), manifest early in the disease course and are associated with an accelerated cognitive and functional decline (Koppel *et al*., 2014b). Given the modest efficacy and adverse effect profile associated with antipsychotic use in AD (Schneider *et al*., 2006), there is a compelling clinical need to elucidate the pathophysiology of psychotic symptoms and identify novel therapeutic targets (Geda *et al*., 2013). There is clear evidence that the psychosis syndrome (delusions and/or hallucinations) (Jeste and Finkel, 2000) represents a distinct endophenotype of AD, with a heritability of around 60% (DeMichele‐Sweet and Sweet, 2010). Attempts to further categorise symptoms using factor analytical approaches have identified two broad subtypes (Cook *et al*., 2003): a ‘paranoid’ subtype, which includes delusions of persecution and/or abandonment, and a ‘misidentification’ subtype, characterised by the presence of misidentification phenomena and/or hallucinations. Research into the phenotypic correlates of psychotic symptoms (predominantly delusions) has provided some evidence to support this classification (Ismail *et al*., 2011; Reeves *et al*., 2012), as the misidentification but *not* paranoid subtype has been associated with more marked AD (neurofibrillary tangle) pathology postmortem (Ferman *et al*., 2013; Forstl *et al*., 1994; Mukaetova‐Ladinska *et al*., 1993) and more global cognitive deficits (indexed by lower mini‐mental state examination (MMSE) scores), in clinical studies (Perez‐Madrinan *et al*., 2004; Reeves *et al*., 2012).

Establishing the neuropsychological profile of psychotic symptoms, and establishing any subtype dependency in relation to the cognitive phenotype, could help to localise discrete functional networks. There is consistent evidence of greater executive and/or ‘frontal’ dysfunction in AD patients with psychotic symptoms (Koppel *et al*., 2014b; Lee *et al*., 2009; Lee *et al*., 2007; Paulsen *et al*., 2000a; Paulsen *et al*., 2000b), including patients who present solely with persecutory delusions (Nagata *et al*., 2009). Inconsistent findings have however been reported across other cognitive domains (Reeves *et al*., 2012). Differences in the criteria used to define psychotic symptoms and a lack of uniformity in the treatment of potential confounding factors (e.g., age, MMSE, educational level, psychotropic medication and affective symptoms) may be partly responsible for these discrepancies. It is also possible that the neuropsychological tests used were not sufficiently sensitive measures of the cognitive factors associated with psychotic symptoms in AD.

In a previously published study, which used positron emission tomography to investigate cognitive, behavioural and motor correlates of striatal dopaminergic function in AD, increased striatal D2/3 receptor availability was associated with the presence of psychotic symptoms, which were predominantly persecutory delusions (Reeves *et al*., 2009). These findings are comparable with data from patients with schizophrenia and consistent with shared theories regarding the involvement of corticostriatal dopaminergic networks in the origins of delusions (Coltheart, 2010; Corlett *et al*., 2010; White and Cummings, 1996). In the same sample, motor speed and sustained visual attention (Reeves *et al*., 2010) correlated significantly with D2/3 receptor availability, but the sample size was too small to meaningfully compare performance in psychotic and nonpsychotic groups. Given the preponderance of persecutory delusions in the sample, it is unclear to what extent these tests might be cognitive markers of the psychosis endophenotype or, more specifically, the paranoid subtype. The current study aimed to investigate the neuropsychological correlates of psychotic symptoms in psychotropic naive AD patients and to test the following hypotheses:

1. Performance on tests of motor speed and sustained visual attention previously shown to correlate with striatal dopaminergic function would differ between psychotic and nonpsychotic AD patients.
2. When separated on the basis of paranoid (persecutory) and misidentification (misidentification phenomena and/or hallucinations) subtypes, differences in performance between psychotic and nonpsychotic patients would be largely accounted for by the paranoid subtype.

A secondary aim was to establish the neuropsychological profile of psychotic symptoms in AD and similarly investigate subtype dependency of any significant findings.

## Methods

### Sample

Eighty patients with probable late‐onset AD (McKhann *et al*., 1984) were recruited from memory services (*n* = 66) and older adult community mental health teams (*n* = 11) within the South London and Maudsley NHS Foundation Trust (SLaM) and a Dementia Care Register, funded by the National Institute for Health Research (NIHR) Biomedical Research Centre (*n* = 3). Exclusion criteria included current or past history of neurological or psychiatric illness, prescription of psychotropic medication (including antipsychotics, antidepressants and anxiolytics), presence of parkinsonian symptoms (bradykinesia, rigidity, facial masking or tremor) or other features suggestive of a diagnosis of Lewy body dementia (DLB) (McKeith *et al*., 2005). The study was approved by The Joint South London and Maudsley and Institute of Psychiatry NHS Research Ethics Committee. Written informed consent was obtained from each participant upon recruitment to the study.

## Study design

### Screening and assessment of psychotic symptoms

A modified version of the Neuropsychiatric Inventory (NPI) (Cummings *et al*., 1994) was used to determine the presence/absence and frequency/severity of past and present psychotic symptoms (delusions and hallucinations domains). Patients were classified as ‘psychotic’ if their carer rated delusions or hallucinations as ever having being present. Similar to our previously published studies (Reeves *et al*., 2009; Reeves *et al*., 2010), no threshold cut‐off was used to define psychotic symptoms. For the subtype analysis, delusions of persecution/abandonment were defined as the paranoid subtype, and delusions that involved misidentification were combined with hallucinations to define the misidentification subtype (shown in Table 1), on the basis of the classification used by Cook *et al*. (Cook *et al*., 2003). Patients who were currently experiencing, or had experienced, both types of symptoms were described as ‘mixed’. The Unified Parkinson's Disease Rating Scale (UPDRS) (Motor Examination) was used to screen for the presence of parkinsonism (cut‐off score of 7) (Ballard *et al*., 1997), the MMSE (Folstein *et al*., 1975) was used to assess global cognitive function (cut‐off score of 10), the National Adult Reading Test (Nelson, 1982) provided an estimation of premorbid intellectual functioning and the Geriatric Depression Scale‐15 (Yesavage *et al*., 1982) was used to exclude participants with clinically relevant depressive symptoms (cut‐off score of 6). Medication status (prescription of cholinesterase inhibitors and/or memantine) was documented at the time of screening and controlled for in all statistical analyses.

**Table 1:** Description and classification of psychotic symptoms (*n* = 34): Neuropsychiatric Inventory (NPI)

Domain	Content^a^	Ever experienced *n* (%)	Currently experienced *n* (%)	Total^b^
Delusions	In danger/others are planning to hurt him or her ***P***	—	1 (1.4)	
	Others are stealing from him or her ***P***	3 (4.3)	17 (24.3)	20 (28.6)
	Spouse is having an affair ***P***	—	3 (4.3)	
	Family members plan to abandon him or her ***P***	—	5 (7.1)	
	Unwelcome guests are staying in his or her house ***M***	4 (5.7)	6 (8.6)	10 (14.3)
	His or her spouse or others are not who they claim to be ***M***	1 (1.4)	4 (5.7)	5 (7.1)
	His or her house is not his or her own ***M***	2 (2.9)	6 (8.6)	8 (11.4)
	Television/magazine figures are present in his or her home ***M***	—	—	
Hallucinations	He or she can hear voices ***M***	—	2 (2.9)	
	Talks to people who are not there ***M***	—	1 (1.4)	
	Seeing things not seen by others ***M***	1 (1.4)	4 (5.7)	5 (7.1)
	Smells odours not smelled by others	—	—	
	Feel things on his or her skin	—	—	
	Tastes without known cause	—	—	
	Any other unusual sensory experiences	—	—	

***P*** items included in the paranoid subtype.

***M*** Items included in the misidentification subtype.

a Content taken from items listed in the delusions and hallucinations domains of the NPI.

b Ever experienced + currently experienced (only presented if different from currently experienced). Six patients were not currently experiencing psychotic symptoms.

## Neuropsychological tests

### Hypothesis‐driven tests

Tests were chosen because of their previously documented associations with striatal dopaminergic (D2/3 receptor) function (Reeves *et al*., 2010) and included the following: (i) *rapid visual information processing* (*RVP*), a test of visual sustained attention from the Cambridge Neuropsychological Test Automated Battery (CANTAB, Cambridge Cognition, UK) (Robbins *et al*., 1994), which requires participants to press a button whenever a specific three‐digit sequence (‘357’) appears on the screen, over a 3‐min period; (ii) *motor screening* (CANTAB), used as a screening test, to establish whether participants were able to see and touch a white box whenever it appears on the computer screen and previously shown to correlate with D2/3 receptor function (Reeves *et al*., 2010); and (iii) *simple reaction time* (CANTAB), a more accurate measure of response time than the motor screening test.

### Exploratory analysis

In order to establish a full neuropsychological profile of psychotic symptoms in AD, test measures were included for the following domains: executive function (*digit span*, *semantic and phonemic fluency* and *Hayling Sentence Completion Task*), memory (*immediate verbal recall*, *delayed verbal recall*, *delayed verbal recognition*, *delayed visual recall* and *delayed visual recognition*), language (*Boston Naming Test*), constructional praxis (*the clock drawing task* and *a shape drawing task*) and visuoperceptual function [*Visual Object and Space Perception Battery* (VOSP): *incomplete letters*, *object decision*, *cube analysis*, *number location*, *plus a preliminary screening test* (*shape detection*) *to determine whether a participant has sufficient visual acuity to complete the other subtests*]. Tests are described and referenced in Table S1.

### Statistical analyses

All statistical analyses were carried out using SPSS 19 (www.spss.com). Between‐group differences in demographic data were analysed using independent samples *t*‐tests, Mann–Whitney *U* and chi‐squared tests. Data that failed to fulfil the assumption of normality were transformed for subsequent analyses. Analysis of covariance (ANCOVA) was carried out for each of the hypothesis‐driven tests, with age, MMSE score and years of education included as covariates and medication status as a fixed factor. In the exploratory analysis, multivariate analyses (MANCOVAs) were performed where there were multiple dependent variables in each cognitive domain and ANCOVA where there was only one dependent variable. Where a MANCOVA resulted in a significant main effect (*p* < 0.05), data were submitted to separate ANCOVAs and significant differences determined using a Bonferroni‐adjusted alpha level. Fisher's least significant difference test was used to correct for multiple pairwise comparisons in the subtype analysis. Patients with mixed symptoms (paranoid and misidentification) were excluded from the subtype analysis as the number of patients who were able to complete the tests (six out of a total of eight) was too small to meaningfully investigate.

## Results

### Demographic and clinical characteristics

Eighty patients were referred to the study, of which 10 were excluded on the basis of specified exclusion criteria. Of the 70 participants (81.0 + 5.6 years; 32 men; MMSE = 22.2 + 4.9) who participated in the study, 34 (48.6%) of the sample were psychotic: 28 (82%) of the psychotic group were experiencing symptoms at the time of assessment and 6 (8%) had experienced symptoms at some point in the previous 6 months. Psychotic symptoms were relatively mild (delusions domain: mean frequency × severity score = 3.1 + 2.8; hallucinations domain: mean frequency × severity score = 2.3 + 1.7) and are detailed in Table 1. Demographic and clinical data are shown in Table 2. Psychotic patients were older (*t*_68_ = −2.67, *p* = 0.01) and showed more global behavioural disturbance, indexed by total NPI scores (Mann–Whitney *U*, *p* < 0.001) than the nonpsychotic group. There were no significant differences in educational level (*t*_68_ = 0.31, *p* = 0.76), illness duration (*t*_68_ = −0.43, *p* = 0.67) or medication status (*x* ^2^ = 1.19, df = 1, *p* = 0.28), but there was a trend for lower MMSE scores in the psychotic group (Mann–Whitney *U*, *p* = 0.07). There was a trend for higher UPDRS scores (Mann–Whitney *U*, *p* = 0.07) in psychotic patients, largely accounted for by a score on 3 in a single participant who presented solely with delusions.

**Table 2:** Demographic and clinical characteristics of psychotic and nonpsychotic patients

	Nonpsychotic (*n* = 36)	Psychotic (*n* = 34)	Test_df_, *p*‐value
Mean (SD; range) age (years)	79.5 (5.5; 67–90)	82.8 (4.8; 75–93)	*t*_68_ = −2.67, *p* = 0.01
Number (%) men	16.4 (4.4)	16.4 (1.1)	*x* ^2^ = 0.05, df = 1, *p* = 0.82
Mean (SD; range) duration of illness	1.8 (1.3; 0.5–4.4)	2.0 (1.66; 0.1–5.6)	*t*_68_ = −0.43, *p* = 0.67
Mean (SD; range) years of education	10.0 (1.8; 6–13)	9.9 (1.4;7–13)	*t*_68_ = 0.31, *p* = 0.76
Mean (SD; range) MMSE	23.5 (3.7; 14–28)	20.9 (5.7; 10–28)	Mann–Whitney *U*, *p* = 0.07
Number (%) prescribed cholinesterase inhibitor and/or memantine	32 (88.9)	27 (79.4)	*x* ^2^ = 1.19, df = 1, *p* = 0.28
Mean (SD; range) UPDRS score	0	0.3 (0.6; 0–3)	Mann–Whitney *U*, *p* = 0.07
Mean (SD; range) NPI: Total	4.2 (4.9; 0–17)	15.5 (14.1; 1–73)	Mann–Whitney *U*, *p* < 0.001
Mean (SD; range) NPI: Total (excluding delusions and hallucinations domains)	4.2 (4.9; 0–17)	12.5 (12.5; 0–61)	Mann–Whitney *U*, *p* < 0.001

SD, standard deviation; MMSE, mini‐mental state examination; UPDRS: Unified Parkinson's Disease Rating Scale (Motor Examination); NPI: Neuropsychiatric Inventory.

Mean (SD) MMSE scores across subtypes [nonpsychotic = 23.5 + 3.7; paranoid = 22.1 + 5.3; misidentification = 20.1 + 6.0; mixed = 20.0 (6.2) (*F*_3,69_ = 2.25, *p* = 0.09)].

### Hypothesis‐driven analysis

Table 3 describes and compares performance on tests of motor speed and sustained visual attention in psychotic and nonpsychotic patients. There was no significant effect of psychotic symptoms on motor speed, indexed by motor latency and simple reaction time (*F*_1,64_ = 0.03, *p* = 0.87; *F*_1,64_ = 0.62, *p* = 0.43, respectively). There was a significant main effect of psychotic symptoms on the RVP task (*F*_1,58_ = 5.94, *p* = 0.02, *η*_p_^2^ = 0.09), indicating fewer correct responses in the psychotic group. The subtype analysis (nonpsychotic, paranoid and misidentification) similarly showed significant between‐group differences (*F*_2,51_ = 3.94, *p* = 0.03, *η*_p_ **^2^** = 0.13). Post‐hoc pairwise comparisons showed that these differences were largely explained by poorer performance in the misidentification compared with the nonpsychotic group (*p* = 0.01). No other between‐group differences were found.

**Table 3:** Motor speed and rapid visual processing (RVP) in psychotic and nonpsychotic patients

Global Analysis	Nonpsychotic (*n* = 36)	Psychotic (*n* = 34)		*F*_df_, *p*	*η*_p_^2^
Motor latency (s)	1.4 (0.6)	1.5 (0.6)		*F*_1,64_ = 0.03, *p* = 0.87	<0.001
Simple reaction time (s)	0.4 (0.1)	0.5 (0.1)		*F*_1,64_ = 0.62, *p* = 0.43	0.01
RVP: number of correct responses^a^	19.1 (4.2)	16.4 (4.1)		*F*_1,58_ = 5.94, *p* = 0.02	0.09
Subtype analysis	Nonpsychotic (*n* = 34)	Paranoid (*n* = 13)	Misidentification (*n* = 11)	*F*_df_, *p*	
RVP	19.1 (4.2)^b^	16.6 (4.4)	14.7 (4.2)	*F*_2,51_ = 3.94, *p* = 0.03	0.13

Separate analyses of covariance were carried out on each performance measure, adjusting for age, mini‐mental state examination (x^4^) and educational level. Medication status was included as a fixed factor.

a Four psychotic and two nonpsychotic patients were unable to complete the RVP task.

b Post‐hoc pairwise comparison showed significant difference (*p* = 0.01) between the misidentification and nonpsychotic group.

### Exploratory analysis

Table 4 describes and compares performance in psychotic and nonpsychotic patients across a range of cognitive domains, using MANCOVA/ANCOVA and controlling for potential confounding variables. There was a significant main effect of psychotic symptoms on visuoperceptual performance, which included four components of the VOSP (*F*_4,60_ = 3.75, *p* = 0.009, *η*_p_^2^ = 0.20). ANCOVAs of individual VOSP components (using a Bonferroni‐adjusted alpha level of *p* < 0.0125) showed a significant effect in relation to incomplete letters (*F*_1,63_ = 8.77, *p* = 0.004, *η*_p_^2^ = 0.12). MANCOVA/ANCOVA was not significant for other cognitive domains. When VOSP performance was separated on the basis of subtype (nonpsychotic, paranoid and misidentification) (Table 5), a significant main effect was observed (*F*_8,106_ = 3.64, *p* = 0.001, *η*_p_^2^ = 0.22). ANCOVA conducted on individual components of the VOSP showed significantly poorer performance on incomplete letters performance (*F*_2,55_ = 10.90, *p* < 0.001, *η*_p_^2^ = 0.28) and more moderate effects in relation to cube analysis, which failed to survive Bonferroni correction (Table 5). Post‐hoc pairwise comparisons showed lower incomplete letters scores in the misidentification group compared with nonpsychotic (*p* < 0.001) and paranoid (*p* = 0.04) groups. No other between‐group differences were found. Given the findings of differential RVP and VOSP performance across subtypes, post‐hoc analyses were conducted across all cognitive domains, to investigate subtype dependency. No significant effects were found (executive function *F*_10,100_ = 0.68, *p* = 0.74; memory *F*_10,104_ = 0.40, *p* = 0.94; language *F*_2,55_ = 0.39, *p* = 0.68; constructional praxis *F*_4,108_ = 0.55, *p* = 0.70).

**Table 4:** Neuropsychological of psychotic symptoms: multivariate analysis across cognitive domains

Cognitive domain	*F*, *p*, *η*_p_^2^	Nonpsychotic (*n* = 36)	Psychotic (*n* = 34)
Executive function	*F*_5,59_ = 0.9, *p* = 0.41, *η*_p_^2^ = 0.08	*n* = 35	*n* = 33
Semantic fluency (number of words)		23.5 (7.5)	19.5 (7.0)
Phonemic fluency (number of words)		26.7 (12.3)	23.0 (12.1)
Digit span		13.6 (2.9)	12.3 (2.6)
Hayling inhibition time (ms)		126.8 (65.2)	112.9 (53.0)
Hayling total errors		9.6 (3.7)	10.8 (3.5)
Memory	*F*_5,60_ = 0.85, *p* = 0.52, *η*_p_^2^ = 0.07	*n* = 36	*n* = 34
Immediate verbal recall		10.2 (3.2)	11.2 (3.2)
Delayed verbal recall		1.2 (1.3)	1.3 (1.3)
Delayed visual recall		1.4 (0.4)	1.0 (0.4)
Delayed verbal recognition		15.4 (3.0)	16.2 (3.0)
Delayed visual recognition		17.4 (3.5)	16.9 (3.5)
Language	*F*_1,64_ = 0.36, *p* = 0.55, *η*_p_^2^ = 0.01	*n* = 36	*n* = 34
Boston Naming Test		11.5 (2.6)	11.2 (2.6)
Constructional praxis	F_2,62_ F = 1.46, p = 0.24, *η*_p_^2^ = 0.05	n = 35	n = 34
Total praxis score		9.1 (1.9)	8.3 (1.9)
Clock drawing task (scale 1–6)		3.2 (1.3)	3.5 (1.3)
Visuospatial perception^a^	*F*_4,60_ = 3.75, *p* = 0.009, *η*_p_^2^ = 0.20	*n* = 36	*n* = 33
Incomplete letters	*F*_1,63_ = 8.77, *p* = 0.004, *η*_p_^2^ = 0.12	18.2 (2.9)	15.5 (4.5)
Object decision	*F*_1,63_ = 3.60, *p* = 0.06, *η*_p_^2^ = 0.05	15.5 (2.8)	13.7 (3.0)
Number location	*F*_1,63_ = 1.56, *p* = 0.22, *η*_p_^2^ = 0.02	7.5 (2.5)	6.6 (3.1)
Cube analysis	*F*_1,63_ = 0.02, *p* = 0.89, *η*_p_^2^ < 0.001	7.0 (2.5)	6.8 (2.7)

Mean (SD) values are shown. Transformations were as follows: delayed visual recall = log_10_(*x* + 1); incomplete letters = *x* ^2^.

*F* ratio, *p*‐value and *η*_p_^2^ values are presented for each multivariate analysis of covariance, adjusting for age, educational level and mini‐mental state examination (x^4^). Medication status was included as a fixed factor.

a *F*, *p* and *η*_p_^2^ values are shown for individual analysis of covariance (Bonferroni‐adjusted alpha level of *p* < 0.0125 was applied).

**Table 5:** Rapid visual processing and visual object and space perception battery across subtypes

Neuropsychological test	Nonpsychotic (*n* = 36)	Paranoid (*n* = 14)	Misidentification (*n* = 12)	*F*_df_, *p*	*η*_p_^2^
VOSP (MANCOVA)				*F*_8,106_ = 3.64, *p* = 0.001	0.22
Incomplete letters	18.2 (1.4)*	16.6 (2.9)**	12.8 (2.2)	*F*_2,55_ = 10.90, *p* < 0.001	0.28
Object decision	15.5 (2.8)	13.3 (3.4)	14.2 (1.7)	*F*_2,55_ = 2.21, *p* = 0.12	0.07
Number location	7.5 (2.5)	6.6 (3.2)	5.3 (3.1)	*F*_2,55_ = 1.87, *p* = 0.16	0.06
Cube analysis	7.0 (2.5)	7.8 (2.0)	4.9 (3.1)	*F*_2,55_ = 3.92, *p* = 0.03	0.13

VOSP, Visual Object and Space Perception; MANCOVA, multivariate analysis of covariance.

Mean (standard deviation) scores for test performance measures are shown. *F* and *p*‐values are presented for MANCOVA/analysis of covariance, after adjusting for age, mini‐mental state examination (x^4^) and educational level. Medication status was included as a fixed factor.

Bonferroni‐corrected alpha level of *p* < 0.0125 was applied to analysis of covariance for individual VOSP components.

Post‐hoc pairwise comparisons show significant differences compared with the misidentification subtype:

Post‐hoc subtype analysis was not significant for other cognitive domains (executive function *F*_10,100_ = 0.68, *p* = 0.74; memory *F*_10,104_ = 0.40, *p* = 0.94; language *F*_2,55_ = 0.39, *p* = 0.68; constructional praxis *F*_4,108_ = 0.55, *p* = 0.70).

* *p* < 0.001.

** *p* = 0.04.

## Discussion

The primary aim of the study was to establish if tests previously shown to correlate with striatal dopaminergic function would differentiate between psychotic and nonpsychotic AD patients and to investigate subtype dependency of any significant findings. The finding of impaired accuracy of RVP performance in psychotic patients is consistent with our previous study, where increased striatal D2/3 availability was associated both with poorer RVP performance and the presence of psychotic symptoms (Reeves *et al*., 2009; Reeves *et al*., 2010). Our findings are also comparable with data on young adults with schizophrenia spectrum disorders (Cattapan‐Ludewig *et al*., 2005; Hilti *et al*., 2010a) and their nonaffected first degree relatives (Hilti *et al*., 2010b), in whom reduced RVP accuracy has been described as a phenotypic marker, reflecting ‘impaired context representation’ (Hilti *et al*., 2010b). The fact that such differences were detectable in patients with predominantly mild symptoms, some of whom were not experiencing symptoms at the time of testing, and who would not fulfil criteria for the psychosis syndrome (Jeste and Finkel, 2000) suggests that impaired RVP accuracy may similarly be a trait marker of psychosis proneness in AD.

Contrary to our prediction, there were no differences between psychotic and nonpsychotic patients in measures of motor speed, which correlated highly with dopaminergic function in our previous study. One possible explanation for this is the differential involvement of striatal subregions in attentional and motor function. In our previous study, RVP performance was associated with D2/3 receptor availability in the associative striatum (caudate), a region that is functionally connected with the dorsolateral prefrontal cortex and has a key role in cognitive control and belief evaluation: In contrast, motor latency scores were correlated with D2/3 receptor availability in the sensorimotor striatum, which forms part of a functional network primarily involved in movement coordination (Reeves *et al*., 2009; Reeves *et al*., 2010).

The findings of the subtype analysis were at odds with our original prediction, as the observed differences in RVP scores were explained by poorer performance in the *misidentification* subtype compared with nonpsychotic patients. These differences were not explained by the trend towards lower MMSE scores in the misidentification group, as MMSE was controlled for in the subtype analysis. The RVP has complex task requirements, including visual recognition (the ability to correctly identify numeric stimuli), selective and sustained attention and working memory (holding a 3‐digit sequence ‘online’). Functional imaging studies have shown activation of a frontoparietal attentional network during RVP performance and, as stimulus speed is increased to make performance more effortful, coactivation of regions involved in visual processing (fusiform gyrus and lateral occipital cortex) (Coull *et al*., 1996). Despite our use of an adapted version of the RVP, which requires participants to respond to only a single target sequence, patients generally found the task more difficult than other test measures in the battery (four psychotic and two nonpsychotic patients were unable to complete the test). Within the context of ‘effortful’ RVP performance, it is possible that lower scores in patients with the misidentification subtype are explained by the presence of additional deficits in visual recognition. The findings of the exploratory analysis would support this suggestion, as the misidentification subtype were markedly impaired in their performance on the incomplete letters task compared with nonpsychotic and paranoid groups. Combined, the current findings implicate impaired object processing in the ventral (temporo‐occipital) visual pathway (Mishkin *et al*., 1983) in the misidentification subtype, particularly the lateral occipital cortex, which contributes both to figural completion (ffytche and Zeki, 1996) and RVP performance.

Impaired visual memory (Rey Osterreith) has been previously reported by Perez‐Madrinan *et al*. (2004) in patients with the misidentification subtype. However, the extent of the difference across subtypes did not achieve significance after controlling for MMSE, and interpretation of the findings was further limited by the inclusion of patients with a range of diagnoses including AD, DLB and vascular dementia. Other studies, which have restricted their investigation to AD, have found no differences in visuoperceptual function between psychotic and nonpsychotic patients. These negative findings may be explained by small sample sizes (*n* < 18 in psychotic group) (Lopez *et al*., 1991; Mentis *et al*., 1995; Staff *et al*., 1999; Starkstein *et al*., 1994) or by their use of tests, including Ravens Progre*s*sive Matrices and Visual Form Discrimination, which are more complex in their requirements, and thus less specific for the ventral visual pathway, than the incomplete letters task.

The study was limited by the number of neuropsychological tests included in the battery, which increased the possibility of a type I error in the exploratory analysis. However, this was balanced by the use of multivariate analyses, which reduced the number of required statistical comparisons, and by initially restricting the analysis of subtype dependency to performance measures that differed significantly between psychotic and nonpsychotic groups. The extent of the difference in incomplete letters scores between psychotic and nonpsychotic patients (which survived Bonferroni correction) and the fact that visual recognition has been implicated by Perez‐Madrinan *et al*. (2004) would argue against this being a spurious finding. This will however need to be replicated in future studies. The co‐occurrence of hallucinations and misidentifications in 7 of the 12 patients in the misidentification subtype meant that it was not possible to explore the specific contribution of misidentification phenomena to the aforementioned findings. This is an important limitation and will need to be further investigated in samples that are sufficiently large to allow a meaningful investigation across all subtypes, including those with mixed symptoms, in the presence and absence of hallucinations.

The decision to have no threshold cut‐off for NPI scores to define delusions or hallucinations was based on the findings of our previous imaging study, in which significantly higher striatal D2/3 receptor availability was found in patients with mild, transient psychotic symptoms, who scored 1 or more on either the delusions or hallucinations domain on the NPI. Although this approach is consistent with a ‘continuum’ model of psychosis, it may have led to the inclusion of ‘false positives’ in the psychotic group.

The study was also limited by the absence of precise data on illness duration, which was documented as time since first presentation to a memory service. As a result, it was not included as a potential confounder in the analysis. A more detailed chronology of the illness, including time to onset of psychotic phenomena, will be required in future studies. We cannot completely rule out the possibility that patients with the misidentification subtype in the current sample represent early, undiagnosed cases of DLB, given the occurrence of visual hallucinations and misidentifications at a relatively mild stage of disease (Ferman *et al*., 2013), the fact that impaired VOSP performance has been previously reported in DLB (Cagnin *et al*., 2013) and the trend towards higher UPDRS scores in the psychotic group. It is therefore possible that early Lewy body pathology is mediating the association between the misidentification subtype and tests of visual attention/visuoperceptual function in the patients studied. Against this is the absence of the broader deficits in visuospatial and visuoperceptual function that are typically observed in DLB (Cagnin *et al*., 2013) and the fact that UPDRS scores were comparable with those previously reported in AD (Cagnin *et al*., 2013).

The absence of significant differences between psychotic and nonpsychotic groups across all other cognitive domains is not wholly surprising, as previous studies have reported largely negative findings after adjusting for MMSE and other potential confounders (Reeves *et al*., 2012). It is possible that the tests used were not sufficiently sensitive markers of the cognitive phenotype, and this is perhaps most relevant in relation to tests of executive function, where scores on verbal (semantic and phonemic) fluency were lower in psychotic patients (Table 4) but not significantly so. Tests that have been specifically designed to detect frontal dysfunction within the context of cognitive impairment, and which have been previously shown to differentiate between psychotic and nonpsychotic AD patients (Nagata *et al*., 2009; Paulsen *et al*., 2000a), may have proved more informative. For example, Nagata *et al*. (2009) found performance deficits on the Frontal Assessment Battery in psychotic AD patients who were almost exclusively of the paranoid subtype. The findings of Nagata *et al*. (2009) and Perez‐Madrinan *et al*. (2004) combined with the current study would argue strongly for an investigation of subtype dependency of phenotypic markers in future studies.

Contemporary models (Coltheart, 2010; Corlett *et al*., 2010) that integrate neuropsychological and associative learning theories have emphasised the importance of perceptual expectations in the pathophysiology of delusions and propose that visuoperceptual deficits, combined with disruption of corticostriatal networks, play an integral role in misidentification delusions. Our findings are also consistent with integrative theories regarding the origins of visual hallucinations within the context of neurodegenerative disease (reviewed by Collerton and Taylor, 2013), which suggest that deficits in attentional control *and* perceptual processing are necessary for visual misperceptions and hallucinations to occur (Collerton *et al*., 2005; Diederich *et al*., 2009; Shine *et al*., 2011). On the basis of the present findings, it is tempting to speculate that psychotic symptoms in AD are underpinned by disruption of the cholinergic/dopaminergic axis within frontostriatal circuits, with additional pathology in the ventral visual pathway in patients with the misidentification subtype. Postmortem studies have shown a greater pathological (neurofibrillary tangle) burden in frontal cortical regions (Farber *et al*., 2000; Koppel *et al*., 2014a; Murray *et al*., 2014). There is also evidence of greater tau pathology in frontal (Ferman *et al*., 2013) and limbic/paralimbic regions (Ferman *et al*., 2013; Forstl *et al*., 1994; Mukaetova‐Ladinska *et al*., 1993) in AD patients with misidentifications, including hippocampal/parahippocampal regions that are functionally connected with the ventral visual pathway. Whether these changes manifest early in the disease course, and contribute to the development of misidentification symptoms, are yet to be established. Positron emission tomography imaging techniques that selectively target tau pathology are a potentially exciting avenue for future research in this area, because they could be used to explore the trajectory of early neuropathological change in AD and its contribution to the psychosis phenotype.

## Conflict of interest

None declared.

**Key points**

- The study aimed to establish the cognitive phenotype of psychotic symptoms in Alzheimer's disease and, where findings were significant, to explore subtype dependency.
- Psychotic patients performed more poorly on the rapid visual processing test of sustained attention and the incomplete letters test from the Visual Object and Space Perception Battery.
- When psychotic patients were separated on the basis of ‘paranoid’ (persecutory) or ‘misidentification’ (misidentifications and/or hallucinations) subtypes, poorer performance was largely explained by the misidentification subtype.
- These findings implicate the ventral visual pathway in the misidentification subtype and warrant further investigation in a larger sample.

## Supporting information

Supporting info item
