# Supplementary material for: Cognitive phenotype of psychotic symptoms in Alzheimer's disease: evidence for impaired visuoperceptual function in the misidentification subtype
Source: Int J Geriatr Psychiatry. 2015 Mar 24;30(12):1147–55. doi: 10.1002/gps.4265 (PMC4988507; doi:10.1002/gps.4265)
Supplement: Supplementary file 1 — Supporting info item [file GPS-30-1147-s001.doc]

| **Sup Supplementary Table S1: Neuropsychological Test Battery** | | | |
| --- | --- | --- | --- |
| **Cognitive Domain** | **Name of test** | **Task description** | **Outcome measure** |
| **Memory** |  |  |  |
| Morris *et al*., 1989 | Immediate verbal recall: Immediate Word List | From the CERAD: Participants read aloud a list of 10 words, after which they are asked to immediately recall as many words as they can remember. They are shown the word list 3 times in total. | Total number of words recalled over the 3 trials |
| Morris *et al*., 1989 | Delayed verbal recall:  Word list recall | From the CERAD: Participants are asked to recall as many words as they can remember, after a delay. | Number of words correctly recalled |
| Morris *et al*., 1989 | Delayed verbal recognition: Word list recognition | From the CERAD: After a delay, participants are shown words seen earlier, together with 10 new words, and are asked to identify which of the words were shown previously. | Number of words correctly identified as previously present or absent |
| Morris *et al*., 1989 | Delayed visual recall: Constructional praxis recall | From the CERAD: After a delay, participants are required to recall shapes copied earlier. | Accuracy of drawings scored out of a total of 11 |
| Warrington *et al*., 1996 | Delayed visual recognition: Camden Short Recognition Memory Test for Faces | From the Camden Recognition Memory Test: Participants are shown 25 different faces, and asked to judge whether each face is pleasant or unpleasant. They are then shown two pictures per page and asked to identify which of the faces they have seen previously. | Number of faces correctly identified |
| **Executive** |  |  |  |
| Wechsler and Corporation, 1997 | Digit span total | From the Wechsler Adult Intelligence Scale-III: Participants are asked to repeat a string of digits either forwards or in reverse order, ranging from 2-9 numbers. | Total number of sequences correctly repeated |
| Benton, 1976 | Semantic and Phonemic Fluency | Participants are given 1 minute to retrieve as many words as possible that start with a specific letter (C, F, L: Phonemic Fluency) or words that belong to a certain semantic category (Animals, Fruits, Vegetables: Semantic Fluency). | Total number of words |
| Burgess *et al*., 1997 | Hayling Sentence Completion Test | Measures response initiation and response suppression, and involves inhibiting prepotent responses. Participants must complete each sentence with a relevant word in Part 1. In Part 2, the word used to complete the sentence must have no association to the sentence. | Total time to respond in part 2, and total number of errors made |
| **Language** |  |  |  |
| Morris *et al*., 1989 | Boston Naming Test | From the CERAD: Participants are asked to name 15 pictures. | Number of correct responses |
| **Visuoperceptual** |  |  |  |
| Warrington and James, 1991 | Shape Detection (screening test)  1.Incomplete letters  2. Object decision  3. Number location  4. Cube analysis | From the Visuospatial Object Perception Battery (VOSP):  A preliminary screening (Shape Detection) test is carried out to determine whether a participant has sufficient visual and sensory capacity. 1. Participants are asked to identify 20 letters which are 70% obliterated; 2. Participants are presented with 20 arrays, each of which displays 2D silhouettes of 1 real object and 3 distractors, and are asked to point to the real object; 3. Participants are asked to identify the number corresponding to the position of a dot; 4. Participants are asked to count the number of solid bricks illustrated. | Number of correct responses |
| **Praxis** |  |  |  |
| Morris *et al*., 1989 | Constructional Praxis | From the CERAD: Participants are asked to copy 4 pictures of increasing complexity. | Accuracy of drawings scored out of a total of 11 |
| Shulman *et al*., 1986 | Clock Drawing | Participants are presented with a large printed circle. They are asked to draw a clock and to set the hands on the clock to ‘ten past eleven’. | Scored on a scale of 1 (perfect) to 6 (no representation of a clock) |

**Supplementary References**

Benton AL. 1976. *Multilingual aphasia examination*. University of Iowa.

Burgess PW, Shallice T, Company TVT. 1997. *The Hayling and Brixton Tests*. Thames Valley Test Company.

Morris JC, Heyman A, Mohs RC, *et al.* 1989. The Consortium to Establish a Registry for Alzheimer's Disease (CERAD). Part I. Clinical and

Neuropsychological assessment of Alzheimer's disease. *Neurology* **39**:1159-1165.

Shulman KI, Shedletsky R, Silver IL. 1986. The challenge of time: Clock-drawing and cognitive function in the elderly. *Int J Geriatr Psychiatry*

**1**:135-140.

Warrington EK. 1996. *The Camden Memory Tests Manual*. Psychology Press.

Warrington EK, James M. 1991. *The Visual Object And Space Perception Battery: VOSP*: Manual. Harcourt Assessmentccop. 1991.

Wechsler D, Corporation P. 1997. Wais-III, *Wechsler Adult Intelligence Scale*, Third Edition: WMS-III, Wechsler Memory Scale, Third Edition :

Technical Manual. Psychological Corporation.
